# Effects of Class IIa Bacteriocin-Producing *Lactobacillus* Species on Fermentation Quality and Aerobic Stability of Alfalfa Silage

**DOI:** 10.3390/ani10091575

**Published:** 2020-09-03

**Authors:** Fuhou Li, Zitong Ding, Adegbola T. Adesogan, Wencan Ke, Yun Jiang, Jie Bai, Shah Mudassar, Yixin Zhang, Wenkang Huang, Xusheng Guo

**Affiliations:** 1 State Key Laboratory of Grassland Agro-ecosystems, College of Pastoral Agriculture Science and Technology, School of Life Sciences, Probiotics and Biological Feed Research Centre, Lanzhou University, Lanzhou 730000, China; lifh17@lzu.edu.cn (F.L.); dingwr@lzu.edu.cn (Z.D.); kewc12@lzu.edu.cn (W.K.); baij18@lzu.edu.cn (J.B.); Shah17@lzu.edu.cn (S.M.); zhangp16@lzu.edu.cn (Y.Z.); hwk960115@outlook.com (W.H.); 2 Department of Animal Sciences, University of Florida, Gainesville, FL 32611, USA; adesogan@ufl.edu (A.T.A.); jiangyun0110@ufl.edu (Y.J.)

**Keywords:** alfalfa silage, bacteriocin, aerobic stability, lactic acid bacteria, fermentation, spoilage

## Abstract

**Simple Summary:**

Bacteriocins produced by lactic acid bacteria are considered good alternatives for feed antibiotics because of inhibiting spoilage microorganisms in silage and non-drug resistance in animals. Owing to the narrow antibacterial spectrum, class I bacteriocin-producing lactic acid bacteria are considered to have limitations as silage inoculants. The research was conducted to evaluate the effects of two class IIa bacteriocin-producing *Lactobacillus* on silage fermentation, microbial population, chemical composition, and aerobic stability. The strains results showed that class IIa bacteriocin-producing lactic acid bacteria could improve silage fermentation quality, reduce counts of molds and yeasts, and improve aerobic stability to a greater extent than inoculation with *Lactobacillus plantarum* MTD/1, a proven, widely used inoculant, which does not produce bacteriocin. The findings of this research are of great value for current understandings and onwards to conduct further research and for possible practical implementation of class IIa bacteriocin-producing lactic acid bacteria as silage inoculants.

**Abstract:**

The effects of two strains of class IIa bacteriocin-producing lactic acid bacteria, *Lactobacillus delbrueckii* F17 and *Lactobacillus plantarum* (BNCC 336943), or a non-bacteriocin *Lactobacillus plantarum* MTD/1 (NCIMB 40027), on fermentation quality, microbial counts, and aerobic stability of alfalfa silage were investigated. Alfalfa was harvested at the initial flowering stage, wilted to a dry matter concentration of approximately 32%, and chopped to 1 to 2 cm length. Chopped samples were treated with nothing (control, CON), *Lactobacillus delbrueckii* F17 (F17), *Lactobacillus plantarum* (BNCC 336943) (LPB), or *Lactobacillus plantarum* MTD/1 (NCIMB 40027) (LPN), each at an application rate of 1 × 10^6^ colony-forming units/g of fresh weight. Each treatment was ensiled in quadruplicate in vacuum-sealed polyethylene bags packed with 500 g of fresh alfalfa per bag and ensiled at ambient temperature (25 ± 2 °C) for 3, 7, 14, 30, and 60 days. The samples were then subjected to an aerobic stability test after 60 days of ensiling. Compared with the CON silage, the inoculants reduced the pH after 14 days of ensiling. After 60 days, pH was lowest in the LPB-treated silage, followed by the F17 and LPN-treated silages. Inoculation of F17 increased concentrations of lactic acid in silages fermented for 7, 14, 30, and 60 days relative to other treatments, except for the LPN-treated silages ensiled for 30 and 60 days, in which the lactic acid concentrations were similar to that of F17 silage. Application of F17 and LPB decreased the number of yeast and mold relative to CON and LPN-treated silages. Compared with the CON silage, inoculant-treated silages had greater aerobic stability, water-soluble carbohydrate, and crude protein concentrations, and lower neutral detergent fiber, amino acid nitrogen, and ammonia nitrogen concentrations. The LPB-treated silage had the greatest aerobic stability followed by the F17-treated silage. Both class IIa bacteriocin producing inoculants improved alfalfa silage fermentation quality, reduced the growth of yeasts and molds, and improved the aerobic stability of the ensiled forage to a greater extent than the proven LPN inoculant. However, higher crude protein concentration and lower ammonia nitrogen concentration were observed in LPN-treated silage relative to other treatments.

## 1. Introduction

Excessive use of antibiotics has resulted in antibiotic resistance, which is one of the greatest current public health challenges, affecting about 2 million people and killing about 23,000 annually in the United States [1]. This has led to examination of the efficacy of alternative additives that inhibit the growth of undesirable bacteria [2,3]. Extensive research in recent years has revealed the potential of lactic acid bacteria (LAB) as alternatives to feed antibiotics for improving the performance and feed efficiency of animals [4,5,6]. Such LAB additives do not cause drug resistance or leave harmful residues and are environmentally friendly, hence they are widely used in food fermentation and feed processing industries worldwide [7]. The use of LAB to control the growth of spoilage microorganisms is especially important because of their ability to produce bacteriocin, a vital antimicrobial product with biopreservation and antibacterial properties [8,9], which can improve the quality and safety of food and feed [3,10]. In particular, using bacteriocin-producing LAB as silage inoculants may improve silage preservation and quality by exploiting the antibacterial properties of the bacteriocin and those of other compounds that also have antifungal properties such as acetic acid and 1,2-propanediol, which are produced during ensiling by LAB [11,12]. A previous study has shown that inoculating forage with bacteriocin-producing bacteria like *Lactococcus lactis* CECT 539 and *Pediococcus acidilactici* NRRL B-5627 was more effective than non-bacteriocin control at inhibiting the growth of *Listeria monocytogenes* [13]. By application of the pure bacteriocin produced by *Pediococcus acidilactici*, Amado et al. [14] further confirmed that the applied bacteriocin could effectively inhibit the growth of *L. monocytogenes*. Other studies have also shown that bacteriocin-producing LAB improved the fermentation quality of silages [15,16,17] and did not adversely affect in vitro dry matter (DM) digestibility [18].

Bacteriocins from LAB are generally assigned into four major classes according to their structural, physicochemical, and molecular properties [3], with class I and II being the most studied groups [19]. Class I bacteriocins, such as nisin, have been commercialized and are widely used in the food industry as a biopreservative [3]. However, most class I bacteriocins produced by LAB only inhibit gram-positive bacteria, hence its efficacy as a silage inoculant will be limited [20,21]. In contrast, most class IIa bacteriocins exhibit a wide spectrum of antimicrobial action [3,22]. However, to the best of our knowledge, little information is available on the efficacy of class IIa bacteriocin-producing LAB as silage inoculants. Thus, the objective of this study was to examine the effects of class IIa bacteriocin-producing *Lactobacillus* strains on fermentation quality, microbial counts, proteolysis, and aerobic stability of alfalfa silage.

## 2. Materials and Methods

### 2.1. Lactic Acid Bacteria Inoculants and Culture Conditions

The inoculants used in the present study included (1) *Lactobacillus plantarum* (BNCC 336943), a class IIa bacteriocin producing LAB which was purchased from Suzhou Bei Na Chuanglian Biotechnology Co., Ltd., China; (2) *Lactobacillus delbrueckii* F17, a class IIa bacteriocin-producing LAB, isolated and screened from spontaneously fermented yak yogurt originally sampled in the Qinghai-Tibet Plateau (Genbank accession no. MF 062645), this strain was deposited at China General Microbiological Culture Collection Center (accession no. CGMCC 11247) [23]; and (3) a non-bacteriocin producing silage inoculant, *Lactobacillus plantarum* MTD/1 (NCIMB 40027), purchased from Ecosyl Products Ltd., Stokesley, UK [24]. All strains used in this study are homofermentative LAB.

The three strains were stored at −80 °C in deMan, Rogosa, Sharpe (MRS) medium with 10% (*v/v*) dimethyl sulfoxide (DMSO). Prior to the experiment, the strains were grown at least twice at 37 °C for 18 h using 1% (*v/v*) inoculum in MRS broth to increase the vitality of the bacteria. A similar growth rate of the three strains was obtained after 18 h of static cultivation and all the OD600 of the fivefold diluted bacterial solution reached 0.8. Then, the strains were purified with the streak plate method [25] and cultured at least two consecutive times using MRS broth. The purity and morphology of the bacteria were checked using an electron microscope (CX31, Shanghai V-ham photoelectric technology Co. Ltd., Shanghai, China) before the experiment.

### 2.2. Laboratory Alfalfa Silage Preparation

Alfalfa (*Medicago sativa* L.) was grown at Purple Pasture Co., Ltd., Wuhe county of Anhui Province (latitude 33.13° N, longitude 117.87° E, altitude 21 m a.s.l., Anhui, China) and mowed at the initial flowering stage of the first cut. The grass was brought to the laboratory within 30 min and wilted by air drying at ambient temperature to a DM concentration of 321 g/kg FW, and then chopped to approximately 1 to 2 cm segments using a forage cutter (9Z-0.4, Henan Yu Translation Machinery Equipment Co. Ltd., Zhengzhou, China) within 40 min. The crude protein (CP), α-amylase neutral detergent fiber (aNDF), acid detergent fiber (ADF), and water-soluble carbohydrates (WSC) concentrations were 228, 334, 233, and 56 g/kg on a DM basis, respectively. The initial pH was 6.4 and the counts of LAB, yeasts, and molds in wilted forage were 7.8, 5.8, and 5.0 log_10_cfu/g, respectively. Briefly, the chopped forage was randomly divided into 84 sub-samples (about 500 g for each sub-sample) for experimental treatments and for chemical and microbial composition analysis of wilted forage. Four sub-samples were frozen at −20 °C for further analysis. The remaining 80 sub-samples (5 ensiling periods × 4 treatments × 4 replicates) were then randomly assigned to one of the following treatments: (1) CON, control, only treated with distilled water; (2) *L. delbrueckii* F17 (F17); (3) *L. plantarum* (BNCC 336943) (LPB); or (4) *L. plantarum* MTD/1 (NCIMB 40027) (LPN). The application rate of each inoculant was 1 × 10^6^ colony-forming units/g (cfu/g) forage, fresh weight (FW) basis. Four replicates were prepared for each treatment at each ensiling duration, and *L. plantarum* MTD/1 (NCIMB 40027) was used as a positive control. In order to evenly mix the inoculum in the chopped alfalfa, each LAB culture was centrifuged (8000× *g*, 5 min) and resuspended in sterile distilled water to achieve an application rate of 1 × 10^8^ cfu of viable cells/mL (the dose of each inoculant was 10 mL/kg of FW). These inoculants were manually and uniformly sprayed onto random piles of 500 g forage using a mini sprayer (5 mL), and were subsequently mixed thoroughly in an ethanol-disinfected plastic container. For the control group, an equal volume of distilled water was applied under the same conditions. To avoid possible cross-contamination, four different containers were used for the treatments. The treated or untreated forages were packed into polyethylene plastic bags (300 mm × 350 mm; PE food vacuum bag; Dong Guan Ming Xin Packaging Materials Factory, Dongguan, China) and ensiled individually. The mini-silos were vacuum-sealed by a vacuum packaging machine (DZ-500, Xinji Packaging Equipment Co. Ltd., Shenzhen, China) and then stored in an air-conditioned room (25 ± 2 °C) for 3, 7, 14, 30, and 60 d.

### 2.3. Analytical Methods

After the designated ensiling duration, four bags from each treatment were opened. A 100 g sample of silage was immediately taken and frozen (−20 °C) for further analysis. Another 20 g fresh sample was homogenized in 180 mL of deionized water with a juice extractor (JYL-Y15, Joyoung Co. Ltd., Jinan, China) for 30 s. The resulting suspension was then filtered using four layers of sterile medical gauze and used for biochemical analysis. The pH of the filtrate was instantly detected using a glass electrode pH meter (Hanna Instruments Italia Srl, Padova, Italy), then a subsample of the filtrate was acidified using 50% H_2_SO_4_ (w/w) and filtered through a 0.22 μm dialyzer before organic acid (including lactic-, acetic-, propionic-, and butyric acids) analysis. An Agilent HPLC 1260 equipped with a KC-811 column (Shodex; Shimadzu, Kyoto, Japan) was used to quantify the concentrations of organic acids. The oven temperature, flow rate, and detection wavelength of SPD were 50 °C, 1 mL/min and 210 nm, respectively. On day 60, a second subsample of the filtrate from the mini-silos was acidified using 250 g/L (w/vol) trichloroacetic acid at a ratio of 1:4 to precipitate true proteins for at least 60 min and then centrifuged at 18,000× g for 15 min at 4 °C. The resulting supernatant was analyzed for concentrations of non-protein nitrogen (NPN), ammonia nitrogen (NH_3_-N), amino acid nitrogen (AA-N), and water-soluble carbohydrates (WSC). NPN was quantified using the method of Kjeldahl N [26]. NH_3_-N concentration was quantified by the phenol-sodium hypochlorite method [27]. AA-N was measured using ninhydrin-hydrazine sulfate colorimetry [28]. WSC was measured using anthrone-sulfuric acid colorimetry, as described earlier [29].

For the enumeration of LAB, yeasts, and molds in both wilted alfalfa and ensiled forage, the modified method of Reich and Kung [11] was followed. Briefly, 100 mL of autoclaved normal saline (0.85% NaCl) was used to homogenize 10 g of sample for 1 min. Then, 10-fold serial dilutions were performed using autoclaved normal saline. The samples were cultured on MRS agar for 48–72 h at 37 °C and LAB were enumerated. Yeasts and molds were enumerated on spread-plates of potato dextrose agar (PDA) supplemented with chloramphenicol after incubation at 30 °C for 48–72 h. An appropriately diluted plate with 30–300 colonies was used for the enumerations and the data of microbial enumeration were expressed as log_10_cfu/g of fresh forage.

The DM concentrations of the wilted and ensiled forage were measured in a forced-air oven at 65 °C for 72 h. The DM loss was determined by the total DM difference before and after ensiling. After grinding to pass through a 1 mm screen, the dried samples were analyzed for Kjeldahl N [26] and crude protein (CP), which was expressed as Kjeldahl N × 6.25. The concentrations of neutral detergent fiber (NDF) and acid detergent fiber (ADF) were quantified using an ANKOM 2000 fiber analyzer (Ankom Technology, Fairport, NY, USA) according to [30]. The NDF was assayed with heat-stable α-amylase (aNDF) and the aNDF and ADF concentrations were expressed inclusive of residual ash.

### 2.4. Aerobic Stability Measurement

On day 60, silages from each silo were subsampled (about 200 g) without compaction into a 500 mL polythene bottle, mixed thoroughly, and subjected to aerobic stability testing at room temperature (29 ± 1 °C). In order to prevent drying and dust contamination and to allow air penetration, a single layer of plastic film with multiple holes was used to cover the top of each bottle. Thermocouples were inserted into the geometric center of each bottle and connected to a data logger (CR10X, Campbell Scientific, Inc., Logan, UT, USA) to monitor the temperature of each silage mass every 30 min. Aerobic stability was defined as the time it took for the temperature in the silage to rise 2 °C above ambient temperature [24]. An empty bottle was used to record the room temperature.

### 2.5. Statistical Analyses

The experimental design had a 4 × 5 factorial design with four treatments and five ensiling durations. The data for pH, organic acids, and microbial counts were analyzed using the general linear model procedure of SSPS 20.0 (IBM Co., Armonk, NY, USA) according to the following model:

Y_ijk_ = μ + I_i_ +D_j_ + (I × D)_ij_ + ε_ijk_

where Y_ijk_ represents the response variable, μ is the overall mean, I_i_ is the effect of inoculants, D_j_ is the effect of ensiling duration, (I × D)_ij_ is the effect of interaction between inoculants and ensiling duration, and ε_ijk_ is the residual error. The effects of inoculants for each ensiling duration were analyzed using Tukey’s test when at least one of the inoculant × ensiling duration interactions was significant (*p* < 0.05). Chemical composition and aerobic stability of day 60 silage samples were analyzed with a one-way analysis of variance (ANOVA) of SSPS 20.0. Tukey’s test was used for pairwise mean comparison. Significance was declared at *p* < 0.05.

## 3. Results

### 3.1. Fermentation Characteristics of Alfalfa Silages During Ensiling

The inoculants, ensiling periods, and their interaction affected the pH and organic acid concentration of alfalfa silage (*p* < 0.05; Table 1). On ensiling for 3 days, LPB-treated silage had the lowest pH, but, by day 7, LPN-treated silage had the lowest pH followed by LPB and the CON silages. Yet, after ensiling for 14, 30, and 60 days, F17, LPB and LPN silages had lowered (*p* = 0.001) silage pH compared with the CON silage and the lowest pH was consistently in the LPB-treated silages. After 7, 14, 30, and 60 days of ensiling, F17 silages had higher (*p* < 0.001) lactic acid concentrations than other silages, except on days 30 and 60, which was not different from LPN silage. Silages inoculated with LPB had lower lactic acid concentration than the CON on days 7, 14, and 30. Compared with the CON silage, the application of the inoculants led to lower (*p* < 0.05) acetic acid concentration on day 3, with the lowest acetic acid concentrations occurring in F17 and LPN silages. However, by day 7, the CON silage had similar acetic acid concentrations to others, except the F17 silage, which had a lower value. On day 14, 30, and 60, all silages had similar acetic acid concentrations. Nevertheless, the mean of acetic acid concentration was lower in LPB-treated silages than the others. Though treatment with F17 resulted in among the lowest propionic acid concentrations on day 3, treatment with F17 or LPN had a greater (*p* < 0.05) mean of propionic acid concentration compared with CON and LPB-treated silages. On day 60, propionic acid concentrations were also greater (*p* < 0.05) in F17 and LPN silages than the CON silage. Silages inoculated with F17 had greater (*p* < 0.05) ratio of lactic acid to acetic acid than the CON, LPB, as well as LPN on days 3, 14, and 30 and greater lactic acid/acetic acid than the CON and LPB on day 60. Inoculating silage with LPB increased (*p* < 0.05) the ratio of lactic acid to acetic acid on day 3, but reduced (*p* < 0.05) it on days 7 and 14 compared with the CON silage. Silages treated with LPN had similar lactic acid to acetic acid ratio as the CON silage in all ensiling time, except on day 7, which showed a lower value.

### 3.2. Microbial Counts of Alfalfa Silage During Ensiling

There were inoculant × ensiling duration interactions on counts of LAB, yeasts, and molds (*p* < 0.05; Table 2). All inoculated silages had a greater population of LAB than CON silage on days 3, 14, and 60, but had similar values to the CON on days 7 and 30. The mean of LAB population was greatest in the LPB-treated silage. Inoculating silage with LPN did not reduce yeast counts compared with the CON silage on days 3 and 7, but did so on day 14. Meanwhile, F17 and LPB silages had lower (*p* < 0.05) yeast counts compared with the CON silage on days 3, 7, and 14. All inoculated silages had higher yeast counts on day 30 than CON, but yeast was not detected in any of the silage on day 60. On day 3, mold counts were lowest in F17 and LPB treated silages (non-detectable) followed by the CON silage, and the highest was in LPN silages. On day 7, no molds were detected in all the treatments except in LPN-treated silage. Silages treated with LPB and LPN had lower (*p* < 0.05) mold counts on days 14 and 60, but higher on day 30 than the CON and F17 silages. On average, lower (*p* < 0.05) mold counts were observed in F17 and LPB-treated silages compared with the CON and LPN silages (0.88 and 0.73 vs. 2.08 and 2.43 log_10_cfu/g of FW, respectively).

### 3.3. Chemical Composition of Alfalfa Silages Ensiled for 60 Days

The DM concentration was greater (*p* < 0.05) in silages treated with the inoculants than the CON on day 60 with F17-treated silage having greater values than the LPN silage (Table 3). All inoculant treatments reduced (*p* < 0.05) the DM loss of silage compared with the CON. The LPB and LPN-treated silages had the greatest (*p* < 0.05) residual WSC concentration, followed by F17-treated silage; all of these had greater concentrations than the CON silage. The CP concentration was greatest (*p* < 0.05) in LPN-treated silages, intermediate in F17 and LPB, and lowest in the CON silage. Inoculating silage with LPB greatly reduced the NPN concentration compared with CON silage (*p* < 0.05; 621 vs. 499 g/kg DM) and other inoculants. The concentrations of AA-N and NH_3_-N in inoculant-treated silages were lower (*p* < 0.05) than that of the CON silage. All inoculated silages had a lower (*p* < 0.05) NH_3_-N concentration than the CON silage, for which the lowest value was observed in LPN-treated silage, followed by LPB silage. Inoculation of LAB reduced (*p* < 0.05) aNDF concentrations, but only LPB reduced ADF concentration when compared with the CON silage.

### 3.4. Effect of Inoculants on Aerobic Stability of 60 d Alfalfa Silage

**Figure 1:**
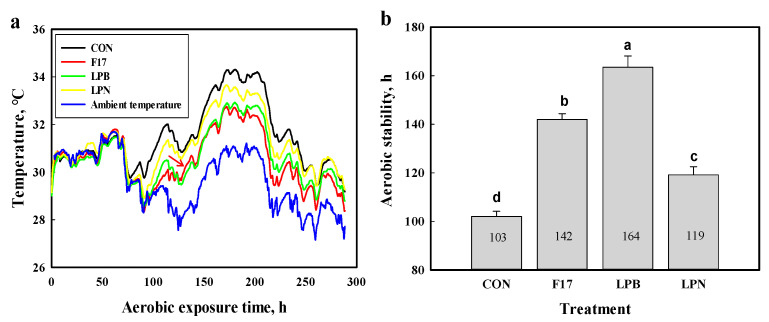
Effects of bacterial inoculants on aerobic stability of alfalfa silage. (**a**), temperature change during the aerobic phase; (**b**), aerobic stability of alfalfa silage treated without or with different inoculants. Treatment: CON, control, no additive; F17, *Lactobacillus delbrueckii* F17; LPB, *Lactobacillus plantarum* (BNCC 336943); LPN, *Lactobacillus plantarum* MTD/1 (NCIMB 40027). Bars with different lowercase letters differed at *p* < 0.05 (n = 4, error bars indicate standard error of the mean).

The changes in temperature and aerobic stability of alfalfa silages during air exposure are presented in Figure 1. The temperature of all silages increased gradually over the first 60 h of aerobic exposure, decreased till about 70 h, and then values changed in a treatment-dependent manner (Figure 1a). Consequently, there were no differences in temperature among treatments during the first 70 h. After 70 h, the temperatures of inoculated alfalfa silages were consistently greater than the ambient temperature, but lower than those of CON silage, except for a few occasions when CON and LPN silages had similar values. All three inoculants improved (*p* < 0.05) the aerobic stability of alfalfa silages compared with the CON (Figure 1b), with the greatest aerobic stability occurring in LPB (164 h), followed by F17 (142 h) and subsequently LPN (119 h) silages.

## 4. Discussion

Alfalfa is typically difficult to ensile because it is a legume which has low DM and WSC concentrations at harvest, as well as a high buffering capacity [31,32]. Excess moisture at harvest in forages can impair silage fermentation [33] and predispose the forage to a clostridial fermentation [34]. Thus, the fresh alfalfa was wilted to 321 g/kg DM in the current study to concentrate the fermentation substrate by removing moisture. The WSC concentration of the forage was 56 g/kg DM, which was sufficient for successful fermentation [35].

The application of LAB inoculants during ensiling is intended to ensure an efficient vigorous fermentation owing to rapid accumulation of lactic acid, which results in a quick reduction in the pH values at early stages of ensiling [36]. However, in the most of the previous studies, only the terminal pH value after ensiling was used to evaluate the effect of LAB inoculants in improving the quality of silage fermentation [37,38]. This does not provide an indication of the dynamic changes caused by inoculants during the silage fermentation process [39]. In the present study, the pH decreased slowly as ensiling progressed. This may be related to hydrolysis of protein in the initial stage of ensiling, which increased the buffering capacity of silage, and thus prevented a rapid reduction in pH [40]. After 14 days of ensiling, a marked reduction in pH was detected in all inoculant-treated silages relative to CON, and the low pH in these groups persisted until the end of the ensiling period. This indicates that, like the *L. plantarum* MTD/1, the proven inoculant, class IIa bacteriocin-producing LAB, F17 and LPB inoculants, improved the preservation and perhaps the quality of alfalfa silage. This finding agrees with those reported by Marcinakova et al. and Silva et al., who used *Enterococcus faecium* EF9296, *Pediococcus acidilactici* 10.6, and *Pediococcus pentosaceus* 6.16 with bacteriocinogenic potential as inoculants [16,17]. In this study, the pHs of the LPB and F17-treated silages were not only lower than that of the CON silage on day 60 of ensiling, they were also lower than that of the LPN-treated silage, which did not contain bacteriocin. The accumulation of lactic acid in F17-inoculated silages caused a marked decline in silage pH after 14 days of ensiling compared with the CON silage. The comparable or lower lactic acid concentrations in the LPB and LPN-treated silages compared with CON silage was opposite with their low pH values throughout the ensiling process and suggested that other acids may be implicated. Specific reasons why concentrations of lactic acid in LPB and LPN-treated silages were relatively low during ensiling are unclear. However, the presence of clostridia or other heterofermentative bacteria may be implicated [17,41,42].

The fact that inoculation with LAB reduced the concentration of acetic acid on day 3 might be because all the inoculants examined in this study are homofermentative bacteria [43]. In particular, the trend for LPB-treated silage, and somewhat for LPN-treated silage, to have the lowest mean acetic acid values was consistent with the widespread use of *L. plantarum* as an inoculant to cause a homolactic fermentation at the expense of producing less acetic acid [44]. Nevertheless, because F17 silage had the greatest mean lactic acid to acetic acid ratio, it was more effective at promoting a homolactic fermentation than the *L. plantarum* inoculants. Compared with the CON silage, the greater propionic acid concentration in F17 inoculated silages fermented for 7 and 60 days and in LPN-treated silages fermented for 30 and 60 days may have resulted from secondary fermentation of lactic acid by enterobacteria and bacteria of the genus *Clostridium* [15,17,34]. The fact that F17 and LPN treated-silages also had the greatest mean propionic acid concentrations was notable, as propionic acid is a potent antifungal acid that can aid aerobic stability [45].

Inoculation increased LAB counts, but the counts in each treatment tended to decrease as ensiling progressed. This might be related to the reduction in the quantity of fermentable carbohydrates remaining as ensiling progressed [13,46]. Yeasts and molds commonly exist in fresh alfalfa and high numbers (>10^5^ cfu/g) adversely affect the preservation of silage and may lead to rapid spoilage when the silage is exposed to air, which may lead to low DM intake and milk production by ruminants [47,48]. Our results showed that the F17 and LPB inoculants reduced mean counts of yeasts and molds to a greater extent than LPN and CON treatments. This was partly because F17 inoculant increased propionic acid concentration, which inhibited the growth of spoilage organisms, and thus enhanced aerobic stability of ensiled forages [45]. Though it is also a strong antifungal acid, concentrations of acetic acid were similar among treatments after 60 days, which indicated that inhibition of yeasts and molds was not owing to acetic acid [49,50]. Rather, the antimicrobial effect that resulted in lower yeast and mold counts for the LPB and F17 silages may have been owing at least in part to their ability to produce bacteriocins [51,52], particularly as the non-bacteriocin LPN inoculant resulted in greater mean yeast and mold counts than the CON silage. It was not clear how the bacteriocin in LPB and F17 contributed to the inhibition of yeast and mold counts since bacteriocins are known to inhibit homologous bacteria, but not fungi [17]. More research is needed to confirm the role of inoculant bacteriocins in inhibiting the growth of spoilage fungi.

Aerobic deterioration of silages is caused by rapid growth of yeasts and molds, which utilize lactic acid and increase the temperature and pH [53]. Although homofermentative LAB inoculation has sometimes led to poorer aerobic stability of silage [54,55], the results of the current study indicated that inoculation of alfalfa silage with bacteriocin-producing LAB strains, F17 and LPB, led to a reduction in temperature after 70 h and exhibited an improvement in aerobic stability during air exposure with greater efficacy than a widely used non-bacteriocin LAB strain LPN (164 and 142 h vs. 119 h). This supports the use of bacteriocin-producing LAB as silage inoculants instead of strains without such proteins and the outcome may be attributable to the inhibition of acetic acid bacteria, which also causes spoilage [56]. Further research should confirm if and how bacteriocins improve the aerobic stability of silage.

Compared with the CON silage, inoculation with homofermentative LAB reduced DM loss and increased the DM and WSC concentrations of silages fermented for 60 days. This might be owing to the lower pH of inoculated silages, which inhibited the growth of undesirable microorganisms and preserved more nutrients during ensiling [57]. The higher WSC concentration in inoculant-treated silages might be owing to the lower pH as well as acid degradation of hemicellulose [58], which is consistent with the lower aNDF concentration in inoculated silages. The lower WSC concentration in F17-treated silage compared with LPB and LPN-treated silages might be because of soluble carbohydrates released by acid or enzymatic hydrolysis of polysaccharides that were used by LAB in F17 treatment for lactic acid production during ensiling [59].

As silage proteolysis adversely affects the efficiency of N utilization by ruminants [60], attempts to reduce the extent of the process are desirable. Extensive proteolysis in silage is mainly caused by residual plant proteolytic enzymes as well as clostridia and enterobacteria [15,17,40]. Proteolytic enzymes hydrolyze plant proteins into NPN, which consists principally of free AA-N, peptide-N, and NH_3_-N [61]. A previous study showed that reducing silage pH was effective at inhibiting proteolysis, because the activity of plant enzymes are reduced under low pH conditions [62]. In addition, the growth of clostridia and enterobacteria is curtailed at low pH of 5 or less [56]. In the present study, silage treated with inoculants resulted in greater CP and lower AA-N concentrations, and this was attributable to the lower pH values resulting from inhibition of proteolysis by the inoculants [63]. The greater reduction of NPN concentration in silage treated with LPB might be related to the lower pH of this treatment compared with those for the CON and F17 silages. Additionally, the highest CP and the lowest NH_3_-N concentrations in LPN-treated silage occurred in silages with lower pH values within the first 7 days of ensiling.

## 5. Conclusions

All silages treated with LAB inoculants improved the fermentation quality, inhibited the growth of yeast and molds, reduced proteolysis, and increased the aerobic stability of alfalfa silage. Inoculation at ensiling of alfalfa with the class IIa bacteriocin-producing LAB strains, *Lactobacillus delbrueckii* F17 and *Lactobacillus plantarum* (BNCC 336943), improved silage fermentation quality, reduced counts of molds and yeasts, and improved the aerobic stability to a greater extent than inoculation with *Lactobacillus plantarum* MTD/1, a proven, widely used inoculant, which does not produce bacteriocin. However, *Lactobacillus plantarum* MTD/1 preserved more available nitrogen, as indicated by the high crude protein concentration and low ammonia nitrogen concentration in silage after 60 days of ensiling.

## Figures and Tables

**Table 1:** Effects of additives and ensiling time on the fermentation characteristics (dry matter (DM) basis) of alfalfa.

Items ^1^	Treatment ^2^	Ensiling Time (d)					Mean	RSD ^4^	*p*-Value ^3^		
		3	7	14	30	60			T	D	T × D
pH	C	5.88 ^aA^	5.84 ^bAB^	5.87 ^aA^	5.73 ^aB^	5.60 ^aC^	5.79 ^a^	0.032	0.001	<0.001	<0.001
	F17	5.76 ^aB^	5.96 ^aA^	5.72 ^bBC^	5.63 ^bC^	5.34 ^cD^	5.68 ^ab^				
	LPB	5.56 ^bB^	5.78 ^bA^	5.56 ^cB^	5.47 ^cB^	5.29 ^dC^	5.53 ^c^				
	LPN	5.72 ^aA^	5.54^cB^	5.73 ^bA^	5.66 ^bA^	5.48 ^bB^	5.62 ^bc^				
LA, g/kg	C	28.5 ^aB^	11.4 ^bC^	35.4 ^bA^	31.6 ^bAB^	32.3 ^bAB^	27.8 ^ab^	0.419	<0.001	<0.001	<0.001
	F17	19.6 ^bD^	15.6 ^aD^	52.4 ^aA^	38.0 ^aC^	44.5 ^aB^	34.0 ^a^				
	LPB	27.8 ^aB^	8.95 ^cC^	24.5 ^cB^	26.1 ^cB^	34.7 ^bA^	24.4 ^b^				
	LPN	16.6 ^cB^	9.19 ^cC^	33.8 ^bA^	36.4 ^aA^	37.7 ^abA^	26.8 ^ab^				
AA, g/kg	C	14.4 ^aB^	8.15 ^bC^	28.2 ^abA^	27.8 ^abA^	28.2 ^A^	21.3 ^a^	0.481	0.023	<0.001	<0.001
	F17	6.43 ^cB^	11.3 ^aB^	32.7 ^aA^	30.3 ^aA^	29.9 ^A^	22.1 ^a^				
	LPB	10.3 ^bC^	8.70 ^bC^	23.4 ^bB^	24.0 ^bB^	30.0 ^A^	19.7 ^b^				
	LPN	7.48 ^cC^	8.04 ^bC^	25.8 ^bB^	31.6 ^aA^	29.1 ^AB^	20.4 ^ab^				
PA, g/kg	C	8.84 ^aC^	3.15 ^bD^	12.5 ^aB^	15.6 ^bA^	15.9 ^cA^	11.1 ^b^	0.571	<0.001	<0.001	<0.001
	F17	4.08 ^cC^	4.76 ^aC^	13.5 ^aB^	15.2 ^bB^	24.9 ^aA^	12.5 ^a^				
	LPB	6.48 ^bD^	3.35 ^bE^	9.62 ^bC^	13.6 ^bB^	19.8 ^bcA^	10.6 ^b^				
	LPN	5.32 ^bcD^	3.67 ^bD^	12.6 ^aC^	18.2 ^aB^	22.9 ^abA^	12.5 ^a^				
LA/AA	C	1.99 ^bA^	1.40 ^aB^	1.27 ^bBC^	1.14 ^bC^	1.14 ^bC^	1.39 ^b^	0.379	<0.001	<0.001	<0.001
	F17	3.05 ^aA^	1.38 ^aCD^	1.60 ^aB^	1.26 ^aD^	1.50 ^aBC^	1.76 ^a^				
	LPB	2.71 ^aA^	1.03 ^bC^	1.05 ^cBC^	1.09 ^bBC^	1.17 ^bB^	1.41 ^b^				
	LPN	2.25 ^bA^	1.15 ^bB^	1.31 ^bB^	1.15 ^bB^	1.31 ^abB^	1.44 ^b^				

^a–c^ Means in the same column differed (*p* < 0.05); ^A–E^ means in the same row differed (*p* < 0.05). ^1^ LA, lactic acid; AA, acetic acid; PA, propionic acid. ^2^ C, control, no additive; F17, *Lactobacillus delbrueckii* F17; LPB, *Lactobacillus plantarum* (BNCC 336943); LPN, *Lactobacillus plantarum* MTD/1 (NCIMB 40027). ^3^ T, treatment; D, ensiling time; T × D, the interaction between treatment and ensiling time. ^4^ RSD, relative standard deviation.

**Table 2:** Effects of additives and ensiling time on the microbiological composition of alfalfa (mean ± RSD).

Items ^1^	Treatment ^2^	Ensiling Time (d)					Mean	RSD ^4^	*p-*Value ^3^		
		3	7	14	30	60			T	D	T × D
LAB	C	9.33 ^bA^	9.31 ^abA^	8.79 ^cB^	8.68 ^abB^	7.80 ^dC^	8.78 ^b^	0.063	<0.001	<0.001	<0.001
log_10_cfu/g	F17	9.57 ^aA^	9.12 ^bB^	9.21 ^aB^	8.80 ^aC^	8.09 ^bD^	8.96 ^b^				
	LPB	9.53 ^aA^	9.59 ^aA^	9.10 ^abB^	8.78 ^aC^	8.13 ^aD^	9.03 ^a^				
	LPN	9.56 ^aA^	9.50 ^abA^	8.99 ^bB^	8.56 ^bC^	8.02 ^cD^	8.93 ^b^				
Yeasts	C	8.05 ^aA^	2.74 ^C^	3.66 ^aB^	ND	ND	2.88 ^b^	0.990	<0.001	<0.001	<0.001
log_10_cfu/g	F17	6.57 ^cA^	ND	3.06 ^bB^	2.40 ^bC^	ND	2.41 ^c^				
	LPB	7.36 ^bA^	ND	2.77 ^bB^	2.70 ^abB^	ND	2.57 ^c^				
	LPN	7.77 ^abA^	2.70 ^B^	1.97 ^cC^	3.05 ^aB^	ND	3.10 ^a^				
Molds	C	4.70 ^bA^	ND	2.13 ^aC^	ND	3.59 ^aB^	2.08 ^b^	1.101	<0.001	<0.001	<0.001
log_10_cfu/g	F17	ND	ND	2.09 ^aB^	ND	2.30 ^bA^	0.88 ^c^				
	LPB	ND	ND	1.70 ^b^	1.95 ^a^	ND	0.73 ^d^				
	LPN	5.59 ^aA^	3.00 ^B^	1.85 ^bC^	1.70 ^bC^	ND	2.43 ^a^				

^a–c^ Means in the same column differed (*p* < 0.05); ^A–D^ means in the same row differed (*p* < 0.05). ^1^ LAB, lactic acid bacteria. ^2^ C, control, no additive; F17, *Lactobacillus delbrueckii* F17; LPB, *Lactobacillus plantarum* (BNCC 336943); LPN, *Lactobacillus plantarum* MTD/1 (NCIMB 40027). ^3^ T, treatment; D, ensiling time; T × D, the interaction between treatment and ensiling time. ND, not detected. ^4^ RSD, relative standard deviation.

**Table 3:** Dry matter and chemical composition of alfalfa silages ensiled for 60 days.

Item ^1^	Treatment (T) ^2^				SEM ^3^	*p*-Value
	C	F17	LPB	LPN		
DM, g/kg	306.57 ^c^	326.33 ^a^	314.93 ^ab^	310.46 ^b^	2.519	0.005
DM loss, g/kg of DM	84.88 ^a^	56.39 ^b^	42.37 ^b^	51.59 ^b^	5.641	0.013
WSC, g/kg of DM	3.86 ^c^	4.35 ^b^	4.54 ^a^	4.65 ^a^	0.092	<0.001
CP, g/kg of DM	202.15 ^c^	215.40 ^b^	212.38 ^b^	222.78 ^a^	2.309	<0.001
NPN, g/kg of TN	621.43 ^a^	632.81 ^a^	499.01 ^b^	607.88 ^a^	16.230	<0.001
AA-N, g/kg of TN	239.59 ^a^	142.08 ^b^	147.57 ^b^	143.20 ^b^	12.582	<0.001
NH_3_-N, g/kg of TN	132.49 ^a^	111.11 ^ab^	84.56 ^bc^	61.93 ^c^	8.095	<0.001
aNDF, g/kg of DM	345.24 ^a^	336.14 ^b^	331.98 ^b^	334.87 ^b^	1.859	0.032
ADF, g/kg of DM	257.55	252.24	247.97	249.79	1.512	0.106

^a–c^ Means in the same row differed (*p* < 0.05). ^1^ FW, fresh weight; DM, dry matter; WSC, water-soluble carbohydrates; CP, crude protein; NPN, non-protein nitrogen; AA-N, amino acid nitrogen; NH_3_-N, ammonia nitrogen; aNDF, neutral detergent fiber, assayed with a heat-stable amylase and expressed inclusive of residual ash; ADF, acid detergent fiber; TN, total nitrogen. ^2^ C, control, no additive; F17, *Lactobacillus delbrueckii* F17; LPB, *Lactobacillus plantarum* (BNCC 336943); LPN, *Lactobacillus plantarum* MTD/1 (NCIMB 40027). ^3^ SEM, standard error of the mean.
